# Supplementary material for: An explanation for origin unwinding in eukaryotes
Source: eLife. 2019 Jul 8;8:e46515. doi: 10.7554/eLife.46515 (PMC6634965; doi:10.7554/eLife.46515)
Supplement: Supplementary file 1. [file elife-46515-supp1.docx]

| **Oligo Name** | **Oligo Sequence** | **Modifications** |
| --- | --- | --- |
| **T20A** | 5’_ACCCTCACACACCACCGCTATGTAATGTCCTAGCAAGCCAGAATTCGGCAGCGTCTTTTTTTTTTTTTTTTTTTTTTTT*T*T*T*T*T*T_3’ | Phosphorothioate linkages between bases are indicated by * |
| **T20A no 3’ tail** | 5’_ACCCTCACACACCACCGCTATGTAATGTCCTAGCAAGCCAGAATTCGGCAGCGTC_3’ |  |
| **T20B** | 5’_GACGCTGCCGAATTCTGGCTTGCTAGGACATTACAGGAATTATACTGTCACCAAC_3’ |  |
| **T20C** | 5’_GTTGGTGACAGTATAATTCCTAGCGGTGGTGTGTGAGGGT_3’ |  |
| **T30A** | 5’_AGACTGCCATACCCTCACACACCACCGCTATGTAATGTCCTAGCAAGCCAGAATTCGGCAGCGTCTTTTTTTTTTTTTTTTTTTTTTTT*T*T*T*T*T*T_3’ | Phosphorothioate linkages between bases are indicated by * |
| **T30A MP** | 5’_AGACTGCCATACCCTCACACACCACCGCTATGTAATGTCCmpTmpAmpGmpCmpAmpAmpGmpCmpCmpAGAATTCGGCAGCGTCTTTTTTTTTTTTTTTTTTTTTTTTT*T*T*T*T*T_3’ | Methylphosphonate linkages between bases are indicated by mp |
| **T30B** | 5’_GACGCTGCCGAATTCTGGCTTGCTAGGACATTACAGGAATTATACTGTCACCAACCACGAGATTT_3’ |  |
| **T30B MP** | 5’_GACGCTGCCGAATTCmpTmpGmpGmpCmpTmpTmpGmpCmpTmpAGGACATTACAGGAATTATACTGTCACCAACCACGAGATTT_3’ | Methylphosphonate linkages between bases are indicated by mp |
| **T30C** | 5’_AAATCTCGTGGTTGGTGACAGTATAATTCCTAGCGGTGGTGTGTGAGGGTATGGCAGTCT_3’ |  |
| **T60A** | 5’_GGCCTGCTCTTATTACCGACACTTACCTTGAGACTGCCATACCCTCACACACCACCGCTATGTAATGTCCTAGCAAGCCAGAATTCGGCAGCGTCTTTTTTTTTTTTTTTTTTTTTTTT*T*T*T*T*T*T_3’ | Phosphorothioate linkages between bases are indicated by * |
| **T60B** | 5’_GACGCTGCCGAATTCTGGCTTGCTAGGACATTACAGGAATTATACTGTCACCAACCACGAGATTTGAAGTAAACCAATTGAGCACATAGCCGCGA_3’ |  |
| **T60C** | 5’_TCGCGGCTATGTGCTCAATTGGTTTACTTCAAATCTCGTGGTTGGTGACAGTATAATTCCTAGCGGTGGTGTGTGAGGGTATGGCAGTCTCAAGGTAAGTGTCGGTAATAAGAGCAGGCC_3’ |  |
| **ORI Top 3’ tail** | 5’_GAAATAGGTTATTACTGAGTAGTATTTATTTAAGTATTGTTTGTGCACTTGCCTGCAGGCCTTTTGAAAAGCAAGCATAAAAGATCTAAACATAAAATCTGTAAAATAACAAGATGTAAAGATAATGCTAAATCATTTGGCTTTTTGATTTTTTTTTTTTTTTTTTTTTTTTTTTTTTTTTTTTTTTTTT_3’ |  |
| **ORI Top 5’ tail** | 5’_TTTTTTTTTTTTTTTTTTTTTTTTTTTTTTGAAATAGGTTATTACTGAGTAGTATTTATTTAAGTATTGTTTGTGCACTTGCCTGCAGGCCTTTTGAAAAGCAAGCATAAAAGATCTAAACATAAAATCTGTAAAATAACAAGATGTAAAGATAATGCTAAATCATTTGGCTTTTTGATT_3’ |  |
| **ORI Top no tail:** | 5’_GAAATAGGTTATTACTGAGTAGTATTTATTTAAGTATTGTTTGTGCACTTGCCTGCAGGCCTTTTGAAAAGCAAGCATAAAAGATCTAAACATAAAATCTGTAAAATAACAAGATGTAAAGATAATGCTAAATCATTTGGCTTTTTGATT_3’ |  |
| **ORI Bottom 3’ tail:** | 5’_AATCAAAAAGCCAAATGATTTAGCATTATCTTTACATCTTGTTATTTTACAGATTTTATGTTTAGATCTTTTATGCTTGCTTTTCAAAAGGCCTGCAGGCAAGTGCACAAACAATACTTAAATAAATACTACTCAGTAATAACCTATTTCTTTTTTTTTTTTTTTTTTTTTTTTTTTTTTTTTTTTTTTT_3’ |  |
| **Ori Bottom 5’ tail:** | 5’_TTTTTTTTTTTTTTTTTTTTTTTTTTTTTTAATCAAAAAGCCAAATGATTTAGCATTATCTTTACATCTTGTTATTTTACAGATTTTATGTTTAGATCTTTTATGCTTGCTTTTCAAAAGGCCTGCAGGCAAGTGCACAAACAATACTTAAATAAATACTACTCAGTAATAACCTATTTC_3’ |  |
| **Ori Bottom no tail:** | 5’_AATCAAAAAGCCAAATGATTTAGCATTATCTTTACATCTTGTTATTTTACAGATTTTATGTTTAGATCTTTTATGCTTGCTTTTCAAAAGGCCTGCAGGCAAGTGCACAAACAATACTTAAATAAATACTACTCAGTAATAACCTATTTC_3’ |  |
| **T50A** | 5’_TATTACCGACACTTACCTTGAGACTGCCATACCCTCACACACCACCGCTATGTAATGTCCTAGCAAGCCAGAATTCGGCAGCGTCTTTTTTTTTTTTTTTTTTTTTTTT*T*T*T*T*T*T_3’ | Phosphorothioate linkages between bases are indicated by * |
| **T50B** | 5’_GACGCTGCCGAATTCTGGCTTGCTAGGACATTACAGGAATTATACTGTCACCTACCACGAGATTTGAAGTAAACCAATTGAGCAC_3’ |  |
| **T50C** | 5’_GTGCTCAATTGGTTTACTTCAAATCTCGTGGTAGGTGACAGTATAATTCCTTTTT3’3’TTTTTTTTTTTTTTTTTTTTTTTTT_5’-C3 spacer | A 3’ to 3’ reverse polarity linkage is indicated by 3’3’. A 3-carbon spacer at the distal 5’ end is also indicated. |
| **T50D** | 5’_TTTTTTTTTTTTTTTTTTTTTTTTTTTTTTTTTTTTTTTTTTTTTTTTTTTAGCGGTGGTGTGTGAGGGTATGGCAGTCTCAAGGTAAGTGTCGGTAATA_3’ |  |
| **T50E** | 5’_GGAATTATACTGTCACCTACCACGAGATTTGAAGTAAACCAATTGAGCAC_3’ |  |
